# Male Genital Schistosomiasis Along the Shoreline of Lake Malawi: Baseline Prevalence and Associated Knowledge, Attitudes and Practices Among Local Fishermen in Mangochi District, Malawi

**DOI:** 10.3389/fpubh.2021.590695

**Published:** 2021-05-21

**Authors:** Sekeleghe A. Kayuni, Mohammad H. Alharbi, Peter Makaula, Fanuel Lampiao, Lazarus Juziwelo, E. James LaCourse, J. Russell Stothard

**Affiliations:** ^1^Department of Tropical Disease Biology, Liverpool School of Tropical Medicine, Liverpool, United Kingdom; ^2^Faculty of Health and Life Sciences, University of Liverpool, Liverpool, United Kingdom; ^3^MASM Medi Clinics Limited, Medical Aid Society of Malawi (MASM), Blantyre, Malawi; ^4^Research for Health, Environment and Development (RHED), Mangochi, Malawi; ^5^Physiology Department, College of Medicine, University of Malawi, Blantyre, Malawi; ^6^National Schistosomiasis and STH Control Programme, Community Health Sciences Unit, Ministry of Health, Lilongwe, Malawi

**Keywords:** MGS, *S. haematobium*, fishermen, semen, Lake Malawi

## Abstract

Male genital schistosomiasis (MGS) is an often-overlooked chronic consequence of urogenital schistosomiasis (UGS) associated with *Schistosoma haematobium* eggs and associated pathologies in the genital system of afflicted men. Despite the first formal description of MGS in 1911 by Madden, its epidemiology, diagnostic testing and case management of today are not well-described. However, since several interactions between MGS and the Human Immunodeficiency Virus (HIV) are known, there is renewed public health interest in MGS across sub-Saharan Africa (SSA). To shed new light upon MGS in Malawi, a longitudinal cohort study was set up among fishermen along the southern shoreline of Lake Malawi in Mangochi District, Malawi, to document its prevalence and assess mens' knowledge, attitudes and practices (KAP). After providing informed written consent, fishermen (*n* = 376) aged 18+ years (median age: 30 years, range: 18–70 years) were recruited and submitted urine and semen for point-of-care (POC) field and laboratory diagnostic parasitological tests. Individual questionnaires were administered to assess their KAP, with praziquantel (PZQ) treatment provided to all participants. Baseline prevalence of MGS (*S. haematobium* eggs in semen) was 10.4% (*n* = 114, median: 5.0 eggs per ml, range: 0.1–30.0) while for UGS (*S. haematobium* eggs in urine) was 17.1% (*n* = 210, median: 2.3 eggs per 10 ml, range: 0.1–186.0) and 3.8% were positive by POC circulating cathodic antigen (POC-CCA), indicative of a *Schistosoma mansoni* infection. Just under 10% of participants reported having experienced symptoms associated with MGS, namely genital or coital pain, or haemospermia. A total of 61.7% reported previous difficulties in accessing PZQ therapy, with 34.8% having received PZQ therapy before. There was a significant correlation between MGS infection and the frequency of fishing in a week (*rho* = −0.25, *n* = 100, *p* = 0.01). In conclusion, MGS is prevalent among local fishermen yet knowledge of the disease is poor. We therefore call for improved availability and accessibility to MGS diagnostics, PZQ treatment within ongoing control interventions. This will improve the lives and reproductive health of men, their partners and communities in this shoreline environment of Lake Malawi.

## Introduction

Schistosomiasis is a prevalent parasitic, neglected tropical disease (NTD) affecting over 220 million people globally, especially in sub-Saharan Africa (SSA) (1, 2). The pathology of this disease in terms of intestinal, liver and urinary presentations is well-known, yet its chronic effect on host genitalia is often ignored or overlooked. Male genital schistosomiasis (MGS) is a gender-specific manifestation of urogenital schistosomiasis (UGS), associated with the presence of *Schistosoma haematobium* eggs and related pathologies in genitalia of men inhabiting or visiting endemic areas in SSA (3, 4). Despite the first reported by Madden (5), the epidemiology, diagnostic testing and case management of MGS are not well-described owing to limited research and diminishing focus over several decades.

Schistosome eggs evoke immunological responses causing granulomata formation and pathological consequences in genital organs from inflammation and fibrosis (6, 7). Men suffering from MGS in endemic areas experience pelvic, coital or ejaculatory pain, abnormal ejaculates, haemospermia, abnormal swelling of genital organs and infertility, which is generally underreported (8, 9). Schistosome eggs and pathologies have also been observed in seminal fluids and tissues of seminal vesicles, spermatic cord, vas deferens and prostrate during parasitological, histopathological and radiological tests (10–13).

By contrast recent global interest in female genital schistosomiasis (FGS) has grown substantially as epidemiological studies have shown increased risk of HIV infection in women having FGS due to characteristic genital mucosal breach, increased abnormal vasculature, inflammatory cells and mediators that facilitate Human Immunodeficiency Virus (HIV) acquisition and replication (14, 15). Similarly, but to a lesser extent, elevated inflammatory cells and mediators have been observed in male genital organs of those with MGS, highlighting similar potential risk for raised viral shedding and increased transmission of HIV (7, 16), both of which may be reduced after PZQ treatment, the mainstay anthelminthic for schistosomiasis (17).

Malawi is one of the SSA countries where both *S. haematobium* and *Schistosoma mansoni* are prevalent and highly focal around most water bodies, especially Lake Malawi (18–20). In addition, HIV prevalence among adult population (15–49 years) is considered high at 10.6% in SSA region, despite the control efforts including provision of antiretroviral treatment (ART) (21–23). Fishermen are one of the high-risk occupational groups in Malawi with higher HIV prevalence, and also with a plausible risk of increased HIV transmission to their sexual partners, if dually infected MGS.

With limited information about the burden of MGS on southern shoreline of Lake Malawi, we recruited a longitudinal study cohort to assess prevalence of MGS among local fishermen dwelling along the southern shoreline of Lake Malawi in Mangochi District. An integral part of this investigation was to assess their knowledge, attitudes and practices related to MGS and HIV infection.

## Materials and Methods

### Study Area, Population, and Sampling

**Figure 1:**
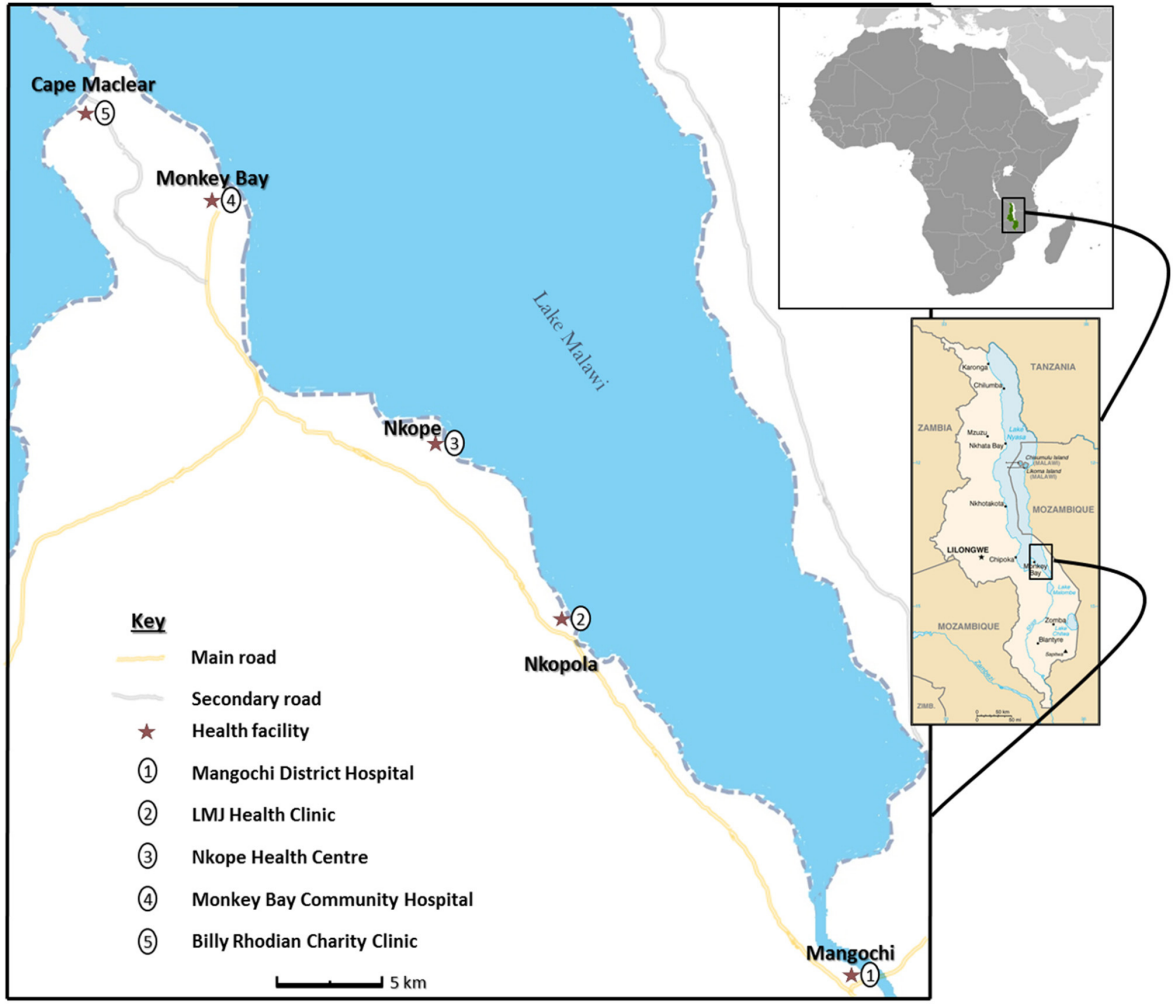
Schematic map of study area showing health facilities along Lake Malawi. (The study map was produced by Dr. Sekeleghe Kayuni (4th August 2019), while the maps of Africa and Malawi were reproduced from the maps at the Central Intelligence Agency (CIA) website, public domain: https://www.cia.gov/library/publications/the-world-factbook/attachments/locator-maps/MI-locator-map.gif and https://www.cia.gov/library/publications/the-world-factbook/attachments/maps/MI-map.gif).

The research study was conducted among fishermen living in fishing communities (villages) identified and selected along southern shoreline of Lake Malawi in Mangochi District, the largest district in southern region of Malawi, from October 2017 to December 2018 (Figure 1). Most of the fishermen in the area live in specific fishing villages, closer to the lake to carry out their routine activities.

Fishermen aged ≥18 years willing to provide written informed consent were eligible to participate in the study. They were asked about results of their recent HIV test within the last 12 months and reported accordingly. A minimum sample size of 275 fishermen was planned to be recruited into the study to measure the current prevalence of MGS, using a desired confidence interval (95%), expected prevalence (20%), and acceptable level of precision (0.05) (24, 25).

### Study Data Collection and Analysis

#### Individual Questionnaires

Briefing about the study was conducted before obtaining written informed consent from the fishermen and recruiting them into the study. Individual questionnaires developed from a previous similar study (26) and piloted in the study area, were administered to the participants, collecting information on demographic, health, hygiene, sanitation, and socio-economic characteristics.

#### Parasitological Analyses

After the questionnaire interviews, the participants were invited to the nearby health facility where they submitted urine, semen and underwent an examination by ultrasonography.

#### Urine Analyses

**Figure 2:**
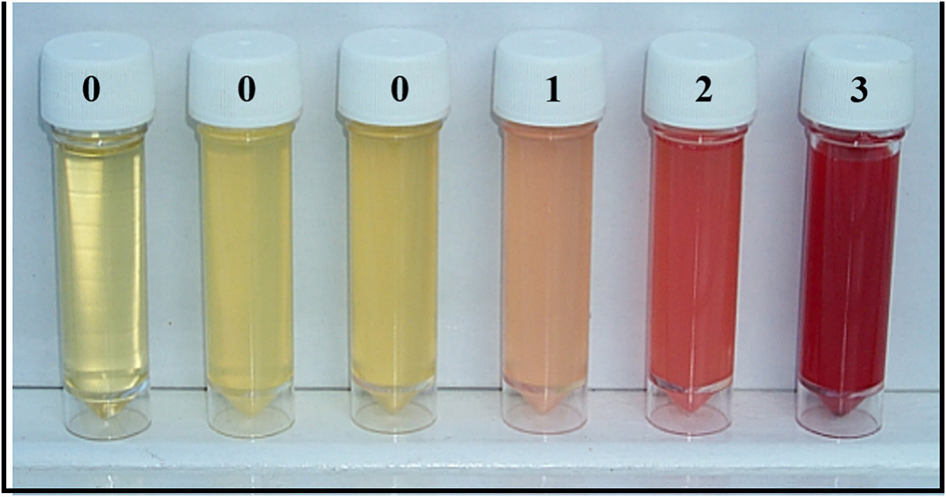
Urine color card for visualization of macrohaematuria.

Participants were provided with a clean 120 ml sample container to submit mid-morning urine. Urine was analyzed immediately for macrohematuria by visual inspection using a urine color card (scores 0, 1, 2, or 3; Figure 2), and then for microhaematuria, leukocytes and proteinuria using reagent strips (Siemens multistix 10G) and scores were recorded in the following categories: negative, trace or positive (graded as +, ++, and +++).

Point-of-care urine circulating cathodic antigen (POC-CCA) test was conducted to assess for possible intestinal infection by *Schistosoma mansoni*, following manufacturer's instructions (Rapid Medical Diagnostics, South Africa; batch no. 171103130) and as described previously (27). Urine volume was measured and recorded accordingly, before conducting filtration following approved standard guidelines to detect schistosome eggs and confirm UGS (28, 29).

The entire volume of urine was filtered through a swinnex plastic holder containing a nylon mesh membrane with 20 μm pores to trap all *S. haematobium* eggs in the sample. The membrane was removed, placed on a standard glass slide and examined under the microscope at ×100 magnification. A drop of Lugol's iodine was added to visualize the eggs distinctly. The number of eggs was calculated by first dividing the total egg number observed by the total volume filtered and then multiplying by 10, to adjust the resultant egg count per 10 ml of urine. High infection intensity for UGS was defined as egg count of ≥50 eggs per 10 ml urine as widely described (29).

#### Semen Microscopic Analysis

Semen was submitted into a transparent, sealable plastic bag to examine at point-of-care for MGS, defined in the study as presence of schistosome eggs in semen. Participants were advised to abstain from coitus for at least 2 days before submitting a semen sample.

The semen bag was placed under ambient temperature on a clean bench surface to allow the semen to liquefy. Thereafter, the semen was pushed gently to one corner of the clear plastic bag before heat-sealing the bag to avoid leakage and to evenly concentrate the semen for easy visualization during microscopy as previously described (30). Direct examination of the semen bag was conducted under a microscope at ×100 magnification to check for schistosome eggs and the presence of leukocytes (31), with results recorded as per ml of ejaculate.

Afterwards, the semen volume was measured and thereafter centrifuged using a benchtop centrifuge with 11 cm radius rotor set at 5,000 rpm for 5 min to separate the seminal plasma (supernatant) from the sediment. The sediment, where schistosome eggs would collect if present, was re-dissolved in 0.5 ml normal saline for wet mount inspection using 2–3 drops and placed on a slide with a coverslip for microscopy, followed by recording of egg count.

### Statistical Analyses

All data collected during the study were screened and quality-controlled before entry into Microsoft Excel and SSPS computer packages. No double data entry was conducted. Screening for errors using descriptive analyses and cleaning were conducted. Frequencies, proportions, medians and ranges of the variables of interest were calculated to define the prevalence of UGS and MGS. Thereafter, the data were explored to further assess the association of different variables related to MGS as well as explore any differences existing between the groups of participants in the study. Non-parametric statistics were used to analyse the data due to its lack of normal distribution.

### Ethical Considerations

Ethical clearance to conduct the study was provided by the National Health Sciences Research Committee (NHSRC, approval number: 1805) of Malawi and Liverpool School of Tropical Medicine (LSTM) Research Ethics Committee (LSTM REC, approval number: 17–018). Utmost privacy and confidentiality were maintained in the study and where necessary, the information was anonymised to protect the identity of the participant. Since this was a test-to-treat study, participants were offered PZQ treatment at the end of each visit before inviting them to the next follow-up study.

## Results

**Figure 3:**
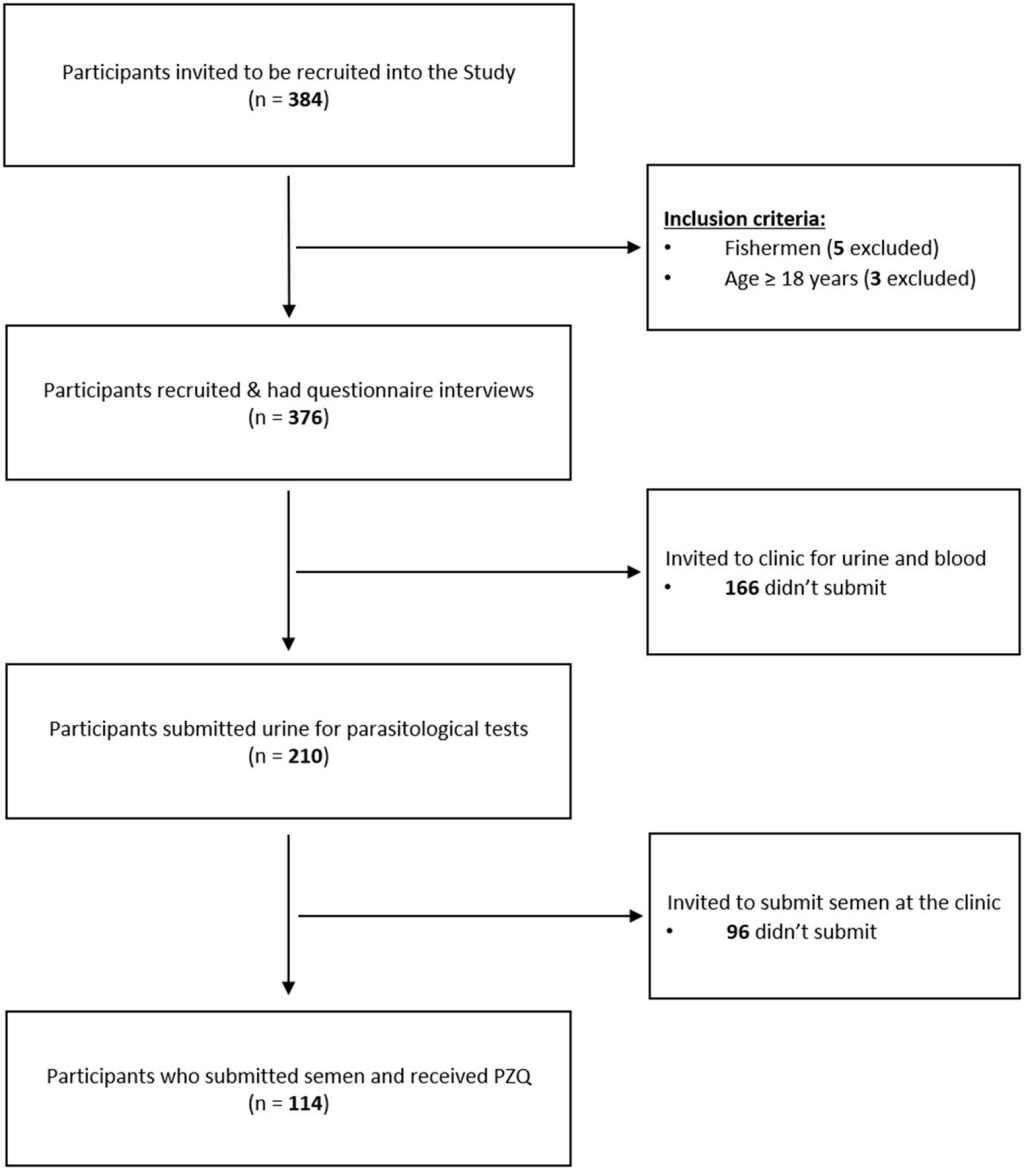
Consort diagram showing the outline of the study.

After the briefing and sensitization about the study, 384 fishermen expressed interest in the study, with 376 recruited and interviewed with questionnaires (Figure 3). Fifty-six participants were HIV positive and taking anti-retroviral therapy (ART) for at least 6 months. The participants came from 39 villages located in two Traditional Authorities (T/A) of Mponda and Nankumba, along the shoreline within study area.

### Demographic Information of the Study Participants

The median age of the participants was 30.0 years with a range of 18.0–70.0 years [Interquartile range (IQR): 13] and their median duration of stay in the fishing village was 20.0 years (IQR: 24.3; Table 1). There was a strong, positive correlation between the age and duration of stay (Spearman's coefficient *rho* = 0.44, *n* = 318, *p* < 0.001). The median weight of the participants was 58.3 kg (IQR: 8.7). For those HIV-positive participants, the median age was 39.5 years (IQR: 17.0), the duration of stay was 24.0 years (IQR: 24) and weight was 56.4 kg (IQR: 8.8) while for HIV-negative participants, the median age, duration of stay and weight were 28.0 years (IQR: 13.0), 19.0 years (IQR: 21.5), and 59.0 kg (IQR: 8.1), respectively. There was a significant difference in the median age between HIV-positive and HIV negative participants (independent samples Mann-Whitney test *U* = 10,917.5, *z* = 5.80, *p* < 0.001).

**Table 1:** Demographic information of the study participants.

	**Variable**	***N***	**Median**	**Range**	**Interquartile range (IQR)**
All participants	Age (years)	376	30.0	18–70	13.0
	Duration in the area (years)		20.0	0.1–70.0	24.3
	Weight (kg)		58.3	43–85	8.7
HIV-negative participants	Age (years)	320	28.0	18–70	13.0
	Duration in the area (years)		19.0	1–70	21.5
	Weight (kg)		59.0	44–85	8.1
HIV-positive participants	Age (years)	56	39.5	21–65	17.0
	Duration in the area (years)		24.0	1–59	24.0
	Weight (kg)		56.4	43–74	8.8
Samples submitted	Urine	210	30.0	18–70	15.0
	Semen	114	29.0	18–67	15.0

Regarding their education status, 11.4% never attended formal schooling, 48.4% did not completed primary school and only 7.7% completed secondary school (Table 2). Of the total participants, 65.7% were married and 67.3% had children regardless of their marital status (range: 1–16 children). There was a positive correlation between participants' age and number of children (*rho* = 0.70, *n* = 260, *p* < 0.001), but negative correlation with education status (*rho* = −0.24, *n* = 370, *p* < 0.001). Apart from fishing, 20% of participants were involved in other activities including farming, business, schooling, and household duties.

**Table 2:** Additional demographic information of the study participants.

	**Variable**	***N***	**Percent (%)**
Level of education	Never went to school	43	11.4
	Didn't complete primary school	182	48.4
	Completed primary school	41	10.9
	Didn't complete secondary school	70	18.6
	Completed secondary school	29	7.7
	Didn't complete tertiary school	4	1.1
	Completed tertiary school	1	0.3
Marital status	Single	83	22.1
	Married	247	65.7
	Co-habiting/engaged	2	0.5
	Divorced	21	5.6
	Other (widowed)	2	0.5
Children	No	64	17.0
	Yes	253	67.3
Other occupation	Farming	2	0.5
	Business	2	0.5
	Household work	69	18.4
	Student	4	1.1
	Unemployed	1	0.3

### Prevalence of UGS and MGS in the Study Cohort

Out of the total recruited participants, 210 submitted urine after questionnaires (55.9%) and 114 submitted semen (30.3%). Forty-three participants on ART submitted urine while 26 submitted semen samples. The median age of participants who submitted urine was 30.0 years (IQR: 15) and for semen was 29.0 years (IQR: 15; Table 1). Urine examination for macrohaematuria using color-score card revealed that most of the urine was clear in appearance (97.1%) while a few samples were cloudy and visually opaque (2.9%).

Upon examination of urine by Siemens multistix showed that 82.4%, 72.9% and 63.8% were negative for leukocyturia, microhematuria, and proteinuria, respectively (Table 3). None of the urine was positive for glucose, suggestive of no glycosuria associated with other diseases.

**Table 3:** Proportion of 210 participants who submitted urine according to results of reagent strip.

**Reagent strip score**	**Leucocytes**	**Blood**	**Protein**
Negative	173 (82.4%)	153 (72.9%)	134 (63.8%)
Trace	14 (6.7%)	28 (13.3%)	29 (13.8%)
+	11 (5.2%)	10 (4.8%)	34 (16.2%)
++	11 (5.2%)	8 (3.8%)	9 (4.3%)
+++	1 (0.5%)	11 (5.2%)	3 (1.4%)
++++	0 (0.0%)	0 (0.0%)	1 (0.5%)

After urine filtration, 36 participants (17.1%) had *S. haematobium* eggs in urine (UGS), their median egg count was 0.9 eggs per 10 ml (IQR: 5.4; Table 4). Only three participants (1.4%) had the highest infection intensity, defined as 50+ eggs per 10 ml of urine (92, 137.8, and 186 eggs). The urine submitted by participants ranged from 10 to 240 ml.

**Table 4:** Parasitological analyses on urine and semen of the study participants.

	**Variable**	***n/N***	**Median**	**Range**	**Interquartile range (IQR)**
All participants	Eggs in urine (per 10 ml)	36/210	0.9	0.1–186.0	5.4
	Eggs in semen (per ml)	12/114	2.9	0.4–30.0	4.3
	Eggs in semen bag (per ml)	12/114	0.8	0.0–9.3	2.5
	Eggs by centrifugation (per ml)	12/114	2.9	0.0–30.0	6.25
HIV-negative participants	Eggs in urine (per 10 ml)	28/166	1.0	0.1–137.8	5.1
	Eggs in semen (per ml)	7/88	3.0	0.8–9.3	4.0
	Eggs in semen bag (per ml)	7/88	2.5	0.5–9.3	3.3
	Eggs by centrifugation (per ml)	7/88	4.7	0.8–6.7	4.3
HIV-positive participants	Eggs in urine (per 10 ml)	8/44	1.4	0.1–186.0	28.9
	Eggs in semen (per ml)	5/26	2.7	0.4–30.0	16.3
	Eggs in semen bag (per ml)	5/26	1.2	0.4–2.0	–
	Eggs by centrifugation (per ml)	5/26	5.0	2.7–30.0	–

Eight (22.2%) of those 36 participants with UGS were on ART, representing 14.3% of HIV-positive participants who submitted urine in the study cohort. There was no significant difference in the urine egg count according to the participants' HIV infection status [median: 1.0 egg, *n* = 21 (Negative); median: 0.1 egg, *n* = 8 (Positive); Mann-Whitney test *U* = 85.0, *z* = 0.05, *p* = 0.96]. In addition, the age of participants did not correlate with UGS infection (*rho* = −0.16, *n* = 36, *p* = 0.35).

Eight participants of those who submitted urine (3.8%, *n* = 210) had a positive POC-CCA test, suggestive of possible *S. mansoni* intestinal schistosomiasis infection. There was a positive correlation between reagent strip scores for leukocytes, blood and proteins with urine egg count (*rho* = 0.23, *p* = 0.001; *rho* = 0.36, *p* < 0.001; and *rho* = 0.25, *p* = 0.001, respectively), while there was no correlation between urine color card with egg count (*rho* = 0.01, *n* = 210, *p* = 0.89).

**Figure 4:**
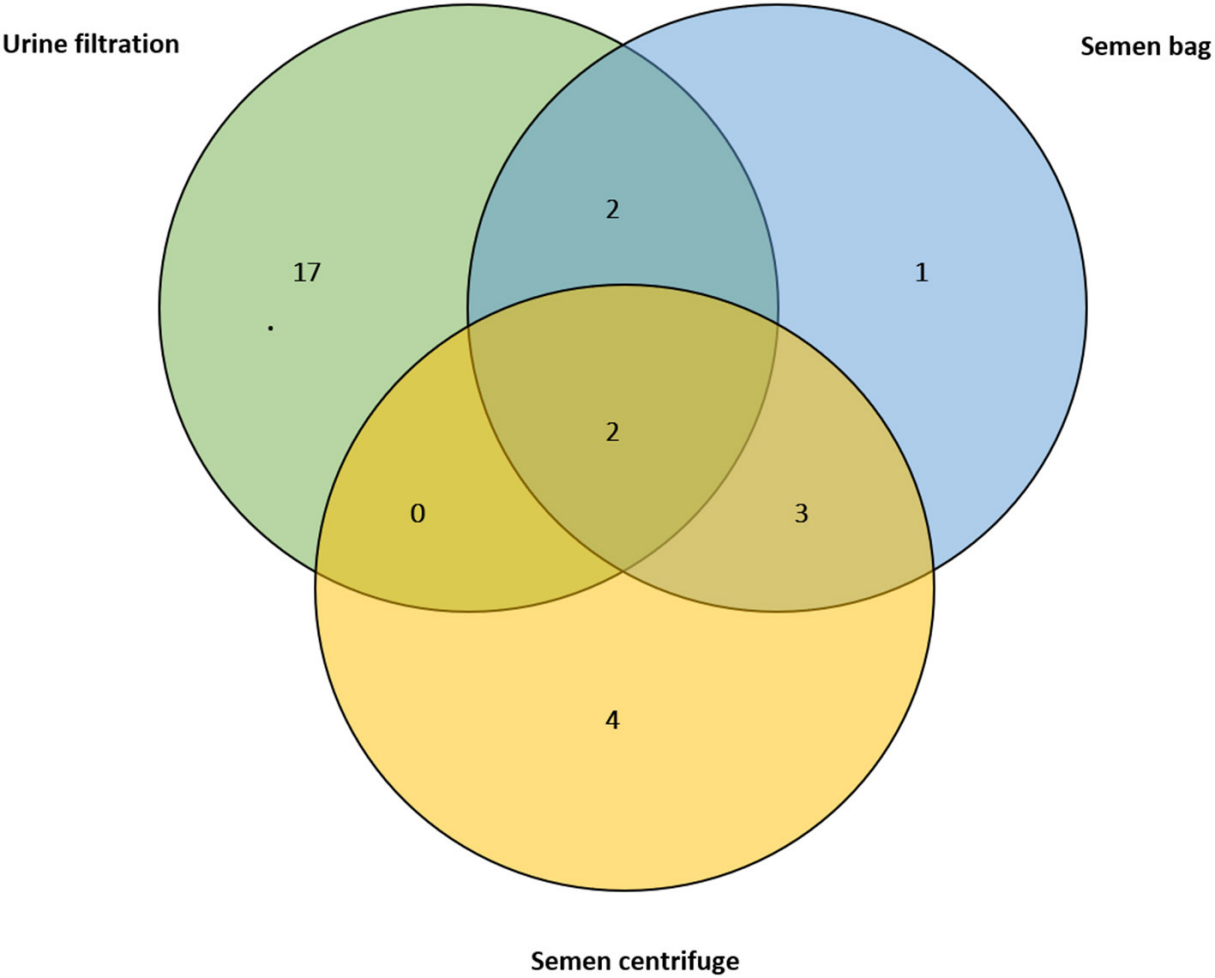
Venn diagram showing positive results of the urine filtration, semen microscopy using bag and centrifugation methods on those participants who submitted semen at baseline (*n* = 114).

For those who submitted semen, 12 (10.4%) had *S. haematobium* eggs in semen (MGS), with median egg count of 2.9 per ml of ejaculate (IQR: 6.3, range: 0.4–30.0 eggs) and seminal volume ranged from 0.1 to 4.5 mL (mean: 1.4 ml). None of the semen had visible blood (haemospermia), or was of abnormal color. The semen bag method identified 8 participants (66.7%) whose mean egg count was 1.7, while the centrifugation method identified 9 participants (75%) with mean of 5.3 eggs, and only five participants (41.7%) were observed to have MGS by both methods simultaneously (Figure 4). There was no statistical difference in the egg count between the methods. Eight participants (66.7%) with MGS had no eggs in urine.

The median age of those with MGS was 46.0 years (IQR: 23.0, range: 18–54) while for those who were MGS negative, it was 29.0 years (IQR: 14, range: 18–67), with statistically significant differences (Mann-Whitney test *U* = 833.0, *z* = 2.04, *p* = 0.04). The ages of participants with MGS correlated significantly with their egg count (*rho* = 0.19, *n* = 114, *p* = 0.001). Furthermore, there was no correlation between semen egg count with urine card score, POC-CCA test results or urine egg count.

Twenty of those participants (17.5%) who submitted semen had leukocytes in their semen, with 13.2% (*n* = 15) had <50 leukocytes, 1.8% (*n* = 2) had 51–100 leukocytes and 2.6% over 100 leukocytes. None of the participants with leukocytes had schistosome eggs in their semen while only 5 participants with *S. haematobium* eggs in urine (UGS), with no correlation and statistical difference (urine: *rho* = 0.035, *n* = 98, *p* = 0.73, Mann-Whitney test *U* = 801.5, *z* = 0.27, *p* = 0.79; semen: *rho* = 0.09, *n* = 98, *p* = 0.37, Mann-Whitney test *U* = 581.5, *z* = 1.01, *p* = 0.31).

### Symptoms and Diseases Reported by Study Participants and Their Spouses

Regarding symptoms related to schistosomiasis, participants reported in their questionnaire interviews among others, change in urine color (47.1%), dysuria (27.6%), frequency (24.9%), haematuria (19.6%), and blood in stool (7.3%) (Table 5).

**Table 5:** Proportion of all 376 participants who reported experiencing symptoms of schistosomiasis including MGS.

	**Symptoms**	***n***	**Percent (%)**
General	Fever	110	30.9
	Weakness	85	22.6
	Abdominal pain	130	34.5
Schistosomiasis	Dysuria	104	27.6
	Urinary frequency	94	24.9
	Urine color change	177	47.1
	Haematuria	74	19.6
	Blood in stool	27	7.3
MGS	Haemospermia	4	1.0
	Pain on coitus	18	4.8
	Pain on ejaculation	12	3.2
	Pain in genital organs	22	5.9

Specifically, for MGS, fewer participants reported to have experienced pain in their genital organs (7.6%), during coitus (4.8%) and on ejaculation (3.2%), haemospermia (1%), with none of those with MGS reporting any of these classical symptoms. In addition, none except one participant with MGS reported having experienced classical symptoms of MGS compared with those with no MGS (Table 6).

**Table 6:** Comparison of 114 participants who reported experiencing symptoms of schistosomiasis according to the MGS infection status.

	**Symptoms**	**MGS-positive** ***(N=12)***		**MGS-negative (*****N=102*****)**	
		***n***	**Percent (%)**	***n***	**Percent (%)**
General	Fever	2	16.7	34	33.3
	Weakness	3	25.0	20	19.6
	Abdominal pain	3	25.0	38	37.3
Schistosomiasis	Dysuria	4	33.3	27	26.5
	Urinary frequency	3	25.0	27	26.5
	Urine color change	6	50.0	41	40.2
	Haematuria	13	11.4	13	12.7
	Blood in stool	1	8.3	5	4.9
MGS	Haemospermia	0	0.0	1	1.0
	Pain on coitus	0	0.0	8	7.8
	Pain on ejaculation	0	0.0	5	4.9
	Pain in genital organs	1	8.3	8	7.8

On the diseases reported, 28.6% had schistosomiasis, malaria (32.1%), diarrhea (31.7%), worm infestation (7.2%) and sexually transmitted infections (STI, 6.4%) (Table 7). On their genital symptoms, two participants thought they were related to STIs (11.2%) and majority were not sure of the cause (66.7%). The participants said that their spouses thought they had STIs (15.4%) and schistosomiasis (7.7%) among others.

**Table 7:** Proportion of all 376 participants who reported the diseases and treatment received in the preceding months before the study.

	**Variable**	***n***	**Percent (%)**
Disease	Malaria	120	32.1
	Diarrhea	119	31.7
	Dysentery	24	6.4
	Worm infestation	27	7.2
	Skin disease	49	13.1
	Sexually transmitted	24	6.4
	infections (STI)		
	Schistosomiasis	107	28.6
Treatment in last 12 months	Antimalarials	134	39.0
	Albendazole	77	22.4
	Praziquantel (PZQ)	123	34.8
Easily accessible to PZQ	No	232	61.7
	Yes	129	34.3

With regards to symptoms reported by the participants' spouses to them, 5.9% had a miscarriage, primary infertility (1.9%) and secondary infertility (2.7%), which have been previously described to be consequences of female genital schistosomiasis (FGS) (Table 8). Furthermore, 2.7 and 0.8% reported to experiencing pain during coitus and bleeding afterwards, respectively, which are also thought to be associated with FGS.

**Table 8:** Proportion of all 376 participants who reported the symptoms and conditions experienced by their spouses in the preceding months before the study.

	**Variable**	***N***	**Percent (%)**
Symptoms	Abdominal pains	68	18.1
	Pain on coitus	10	2.7
	Bleeding after coitus	3	0.8
	Menstrual pains	36	9.6
	Menstrual change	36	9.6
Conditions	Miscarriage	22	5.9
	No children in marriage	10	2.7
	Infertility	7	1.9

Regarding the spouses' symptoms, the majority of participants thought they were due to either normal body functioning (22.4%) or contraceptives from health facility (20.4%), while some thought it was unknown disease (14.3%), pregnancy (8.2%), schistosomiasis (2.0%), and other conditions, with one surprisingly saying, “*I think her blood is bad, doesn't relate well with mine.”* The spouses themselves had similar thoughts of normal body functioning (19.4%), contraceptives (19.4%), pregnancy (8.1%), unknown diseases (6.5%) among others, with one saying, “*I think this is as a result of witchcraft.”*

Most participants expressed their need for assistance with their symptoms and diseases (39.1%), diagnostic testing (13.0%) and treatment (8.7%) of schistosomiasis, and further information on the common diseases in the area (30.4%), namely schistosomiasis, malaria, HIV/AIDS, and contraceptives use. Of particular interest, one participant commented that, “*do not use modern contraceptives from health facility, rather use traditional methods from the village.”*

Praziquantel was accessed by 34.8% of participants in the last 12 months, 22.4% received albendazole, and 61.7% reported that they had difficulties to access treatment for schistosomiasis (PZQ), although there was no significant correlation with MGS in this study.

### Water Contact, Fishing, and Sanitation Information of the Study Participants

The participants admitted to swimming or walking (92.5%), bathing and washing their bodies and clothes (94.1%) in the lake, at least four times in a week. The main reasons for using the lake were because of its convenience to their daily routine since they spend significant amounts of time in the lake (44.9%), and it is clear, fresh and free water (41.9%), which encourages them to be clean (6.4%), while a small proportion reported that the borehole water was salty (1.0%) or located at a far distance (3.7%).

With regards to their fishing, the median duration of fishing was 7.0 years (IQR: 9.3, range: 0.2–60 years) and participants spending an average of 4 days per week on fishing in the lake (IQR: 5, range: 1–7 days; Table 9). There was a strong, positive correlation between participant's age and number of years he has been fishing (*rho* = 0.51, *n* = 298, *p* < 0.001) as well as frequency of fishing in a week (*rho* = 0.14, *n* = 352, *p* = 0.008), highlighting the potential increased risk of exposure to schistosomiasis.

**Table 9:** Proportion of participants who reported on their water contact and fishing history in the Lake in the preceding months before the study.

**Variable**	***N***	**Median**	**Range**	**Interquartile range (IQR)**
Swim/walk in the Lake (times per week)	371	6	1–7	4
Bath/wash in the Lake (times per week)	373	4	1–7	5
Protective wear in the Lake (times per week)	372	5	1–7	4
Fishing in the Lake (number of years)	298	7	0.2–60	9
Frequency of fishing (days per week)	352	3	1–7	5
Fish migration to other areas (times per year)	354	3	1–20	2

Close to two-thirds (66.2%) admitted to fish in other areas, more than 3 times in a year (IQR: 2, range: 1–20), mainly following larger fish catches at a particular time (91.6%), as well as increased availability of fishing boats in specific area (3.8%), following friends migrating to other areas (peer pressure, 3.4%) and better fish prices (1.3%).

Since the lake closes to fishing from December to January every year, in accordance to Malawi government regulations for fish breeding season, most of the participants do other activities including farming (50.4%), casual work, other skilled works (hair-cutting, tailoring, carpentry, building, welding: 7.7%), while some do nothing (22.2%). The duration of fishing was negatively correlated with education status of the participants, which was statistically significant (*rho* = −0.16, *n* = 295, *p* = 0.005).

Only 12.7% use protective wear during their water contact. The reasons for not using protective wear include lack of special wear (17.3%), inconveniencing as they have difficulties working with them (19.7%), fear of drowning due to heavy weight once soaked (26.5%), while some find them not necessary (7.1%) and 10% stated that there are rules against use of protective wear or any heavier clothing when fishing in the lake.

Half of study participants used their home toilet for urination (56.9%), followed by those using the lake (32.3%), both lake and home toilet (9.7%) and only 3 use the bush (0.8%). Similarly, more participants used their home toilet (60.8%) for defecation, some still use the lake (30.8%), both places (9.7%), and the same number used the bush. Thus, indicating the participants do increase the risk of being infected during frequent water contact and transmitting the disease due to their poor sanitary behavior.

Most have home toilet (92.4%), which they used on average 6.3 days in a week (95% C.I.: 6.2–6.5 days), contradicting to their earlier response. For those participants without, they report using the toilet of their parents (50%), neighbor (30%), or drinking bar (10%).

### Multivariate Analysis of Reported Symptoms, Diseases, Water Contact, and Tests Results

Further statistical analyses were conducted to explore the relationship between the MGS infection status and reported symptoms, diseases, water contact and fishing history. There was a significant correlation between MGS infection and the frequency of fishing in a week (*rho* = −0.25, *n* = 100, *p* = 0.01) and fishing in other areas (*rho* = 0.23, *n* = 73, *p* = 0.05), while only the duration of stay in the study site was slightly significant correlated with UGS infection (*rho* = 0.16, *n* = 178, *p* = 0.03).

There was no correlation between infection status and number of days involved in swimming, walking, working, bathing, or washing in the lake per week, use of protective wear in the lake or use of home toilet. However, there was statistical difference in MGS infection status with regards to their use of the lake for bathing or washing (*p* = 0.01), explanation for their use of the lake (*p* = 0.008), and their village of stay (*p* = 0.001).

Furthermore, there was a significant statistical difference in the fishing weekly duration of participants, according to their MGS infection using the Mann-Whitney *U* test (median: 3 days, *n* = 93; median: 1 day, *n* = 7; *U* = 142, *z* = −2.54, *p* = 0.01, *r* = 0.03; respectively), suggesting that those with less number of fishing days were likely to have MGS. There was no difference in the frequency of water contact during swimming, bathing, washing, number of years fishing and frequency of fishing in other areas. All the variables were not statistically different with their UGS infection status. There was no statistical difference in the infection status with the response to the symptoms and diseases experienced by the participants, during and in the months preceding the study.

On the participants' response to PZQ access for schistosomiasis treatment, there was no significant difference in MGS and UGS infection status. Similarly, on the easy access to PZQ treatment for schistosomiasis was not significantly different between those participants with and without MGS, similarly to those who either or not received treatment in the last 12 months preceding the study.

One-way between groups analysis of variance (ANOVA) was conducted to explore the impact of participants' education status on the MGS and UGS infection, which were divided into four groups according to their responses: never went to school, primary education, secondary education, and college education. With no violation of homogeneity of variances, there was no statistically significant difference in UGS and MGS infection among the education status groups of the participants: *F*_(2, 204)_ = 0.20, *p* = 0.82 (UGS), and *F*_(2, 108)_ = 0.07, *p* = 0.92 (MGS). Of the MGS participants in the study, 9 (75%) attended primary education while the remaining 3 (25%) attended secondary education.

### Multivariate Analyses of the Different Diagnostic Tests for Schistosomiasis

On the relationship between the tests conducted in the study, there was no statistical difference between the urine color-card scores and the urine egg counts using Kruskal-Wallis tests (*n* = 210, *p* = 0.89), while there was significant difference in the reagent strip scores and urine schistosome egg counts [*n* = 210, *p* = 0.012 (leukocytes), *p* < 0.001 (blood, protein)]. The reagent strip scores correlated with the semen egg count [*rho* = 0.23, *p* = 0.001 (leukocytes); *rho* = 0.36, *p* < 0.001 (blood); and *rho* = 0.25, *p* = 0.001 (protein)], while the color card scores were not correlated with semen egg counts.

Comparing the urine tests with semen egg results, there was statistical difference between the reagent strip scores for protein and semen egg counts (*n* = 114, *p* = 0.01), with no difference in reagent strip scores for leukocytes, blood and color card scores. When compared, the semen bag and centrifugation methods had a Kappa measure of agreement value was 0.56, with statistical significance (*p* < 0.001). Using semen centrifugation method as a reference test, the sensitivity of semen bag was 55.6% while specificity was 97.1%, illustrate the capability of bag method in MGS testing.

## Discussion

This is the first extensive research study investigating MGS among local inhabitants along the shoreline of Lake Malawi where urogenital schistosomiasis is endemic. Previous reports have been case descriptions of travelers or non-dwellers visiting the lake for recreation or business (32–34).

Despite its first description over a century ago by Madden (5), MGS remains little known, poorly recognized, misdiagnosed and underreported among men in endemic areas such as Lake Malawi shoreline, thereby suffering from its consequences ranging from persistent genital, coital and ejaculatory pain, abnormal ejaculates, haemospermia, swollen organs, and infertility. Coupled with the poor health-seeking behavior of men observed in endemic areas, certainly MGS impacts the male reproductive health negatively, making it an ignored aspect of an NTD and a public health concern in such endemic areas.

Our study population comprised young and middle-aged fishermen who had spent most of their life on the lake shoreline in the fishing communities, as observed from their median age of 30.0 years. This is similar to the national trend of the country population, pegged at 17.6 million in 2018 with over 230,000 men aged ≥18 years in the district and over 70,000 in the two T/A of the study area. In such endemic areas, people become infected as early in life as infants (35) and as they grow, repeated exposure through contact with infested lake water, results in re-infections and progressive development of chronic manifestations of schistosomiasis, including MGS in males (36, 37). Despite some case reports on MGS being in young male children (38, 39), most reports on MGS have been in adult males beyond adolescence, similar to our observation for the mean age of those with MGS being higher and significantly different to those without disease (34).

The prevalence of MGS observed in our study was similar to the assumed prevalence (10.4%), but lower from previous studies in other endemic countries (4, 7). As observed in this study, the majority of MGS participants (66.7%) had no eggs in urine (UGS) which also explains the non-correlation of both tests. Urine filtration has been used as a proxy to diagnosing MGS, due to challenges encountered in semen submission with individual perceptions and cultural myths around the sample (40). However, filtration is known to have low sensitivity and specificity especially when the prevalence starts declining (41), especially with the mass drug administration (MDA) campaigns with PZQ, which the national control programme in Malawi conducts annually. Interestingly, in the study, the scores of the urine reagent multistix strips correlated significantly with MGS, and suggests the need to develop more accessible, affordable, point-of-care sensitive and specific diagnostic tests for MGS (42).

The novel semen bag method described and used in the study showed reasonable sensitivity and specificity when compared to the standard semen examination technique (centrifugation method) which has been routinely used (31). This could serve as the first-line examination tool for semen in diagnosing MGS in endemic areas, owing to the simple availability of the tool, compared to most sample collection tools like non-spermicidal condoms or disposable containers.

In addition, the use of POC-CCA urine tests could assist in determination of intestinal schistosomiasis (27, 41, 43), which happen to be an emerging infections having autochthonous transmission on the shoreline (44). Further investigation of positive men on POC-CCA can elucidate this infection and explain the absence of *S. mansoni* eggs in semen unlike previous reports from other areas (45–47).

With regards to the symptoms, fewer participants reported having the classical symptoms of UGS and specifically MGS, as none of the symptoms here had a significant relationship with MGS infection status. This was similar to their perceptions of the symptoms not related to schistosomiasis but rather other diseases including STI, similarly observed in other studies (26, 48). In previous reports, such classical symptoms such as haemospermia and abnormal ejaculates were observed in naïve individuals visiting endemic areas and displaying early stages of schistosomiasis preceding diagnosis of MGS (33, 49, 50).

On their water contact during bathing, washing or swimming in the lake, there was no correlation observed with MGS infection status which was surprising. However, their frequency of fishing in the lake per week was noted to correlate with the infection, supporting the known fact that repeated exposure to infested water increases the risk of schistosome infection, its intensity and afterward development of MGS, coupled with their low usage of protective wear (37, 51). The mean frequency of fishing was significantly different between the infection statuses which suggest more days of fishing contribute to more exposure which subsequently result in MGS infection.

Furthermore, practices of some participants in using the lake for urination and defecation instead of toilets contribute to continuation of the life cycle because the intermediate snail hosts will be infected with miracidia from their hatched eggs. This does not appear surprising observing that the level of education had no impact on the infection status, since all the MGS participants attended primary and secondary education, although education is supposed to contribute toward modification and transformation of behavior with regards to schistosomiasis (52). There is need for comprehensive health education through diverse available channels like beach committee meetings which involves all fishermen, community radio programs, health meetings among others in order to engage the men on schistosomiasis and MGS in particular. Certainly, there is need to develop local tailor-made water, sanitation and health (WASH) interventions together with the men to address issues of poor hygiene and sanitation observed in the study.

The drop-out of participants in submitting samples, especially semen, limit the generalization of the study results to male populations in the country and endemic region. This could be explained by negative perceptions and myths associated with semen in rural communities, although a previous study examining semen in the district did not encounter such challenges (53). In addition, the rumors of “*blood suckers*” (vampires) visiting and terrorizing the local communities negatively affected the trust and confidence local men had to the study team, thus additional sensitization and discussions were conducted with local traditional and opinion leaders, health workers and police officers (54–57). Also, some participants could be reluctant to submit samples at the health centers, due to poor health-seeking behavior. Similarly, previous studies describing MGS had similar or even lower number of participants submitting semen, highlighting the sensitivities and challenges in such studies. Thus, these valuable results are important if we are to build on and contribute to the current knowledge of MGS in local inhabitants of a schistosomiasis endemic area. Lack of sperm assessment in this study for sperm count, motility and morphology among other andrological parameters which are potentially important aspects of MGS limited its further understanding in fishermen of this endemic area.

In conclusion, male genital schistosomiasis remains a prevalent, poorly recognized manifestation of schistosomiasis, especially UGS, commonly in men with frequent exposure to infested waters in endemic areas like Lake Malawi shoreline. This study illustrates the need for more education on schistosomiasis, specifically MGS among men, to seek timely medical assistance and access PZQ, which is known to have excellent egg-reduction and cure rates of up to 100% (58).

## Data Availability Statement

The raw data supporting the conclusions of this article will be made available by the authors, without undue reservation.

## Ethics Statement

The studies involving human participants were reviewed and approved by National Health Sciences Research Committee of Malawi (NHSRC, approval number: 1805) and Liverpool School of Tropical Medicine (LSTM) Research Ethics Committee, United Kingdom (LSTM REC, approval number: 17-018). The patients/participants provided their written informed consent to participate in this study.

## Author Contributions

SK, EL, and JS conceptualized the study. SK, MA, PM, FL, LJ, EL, and JS conducted the field data collection and analyses. SK drafted the manuscript. All authors contributed to the final version of the manuscript.

## Conflict of Interest

SK was employed by MASM Medi Clinics Limited. The remaining authors declare that the research was conducted in the absence of any commercial or financial relationships that could be construed as a potential conflict of interest.
